# Effect of Urate-Lowering Therapy on All-Cause and Cardiovascular Mortality in Hyperuricemic Patients without Gout: A Case-Matched Cohort Study

**DOI:** 10.1371/journal.pone.0145193

**Published:** 2015-12-18

**Authors:** Jiunn-Horng Chen, Joung-Liang Lan, Chi-Fung Cheng, Wen-Miin Liang, Hsiao-Yi Lin, Gregory J Tsay, Wen-Ting Yeh, Wen-Harn Pan

**Affiliations:** 1 School of Medicine, China Medical University, Taichung, Taiwan; 2 Department of Internal Medicine, China Medical University Hospital, Taichung, Taiwan; 3 Department of Public Health, China Medical University, Taichung, Taiwan; 4 Graduate Institute of Biostatistics, Biostatistics Center, China Medical University, Taichung, Taiwan; 5 School of Medicine, National Yang-Ming University, Taipei, Taiwan; 6 Department of Internal Medicine, Veterans General Hospital, Taipei, Taiwan; 7 Institute of Biomedical Sciences, Academia Sinica, Taipei, Taiwan; 8 Institute of Population Health Sciences, National Health Research Institutes, Miaoli County, Taiwan; University of São Paulo School of Medicine, BRAZIL

## Abstract

**Objectives:**

An increased risk of mortality in patients with hyperuricemia has been reported. We examined (1) the risk of all-cause and cardiovascular disease (CVD) mortality in untreated hyperuricemic patients who did not receive urate-lowering therapy (ULT), and (2) the impact of ULT on mortality risk in patients with hyperuricemia.

**Methods:**

In this retrospective case-matched cohort study during a mean follow-up of 6.4 years, 40,118 Taiwanese individuals aged ≥17 years who had never used ULT and who had never had gout were examined. The mortality rate was compared between 3,088 hyperuricemic patients who did not receive ULT and reference subjects (no hyperuricemia, no gout, no ULT) matched for age and sex (1:3 hyperuricemic patients/reference subjects), and between 1,024 hyperuricemic patients who received ULT and 1,024 hyperuricemic patients who did not receive ULT (matched 1:1 based on their propensity score and the index date of ULT prescription). Cox proportional hazard modeling was used to estimate the respective risk of all-cause and CVD (ICD-9 code 390–459) mortality.

**Results:**

After adjustment, hyperuricemic patients who did not receive ULT had increased risks of all-cause (hazard ratio, 1.24; 95% confidence interval, 0.97–1.59) and CVD (2.13; 1.34–3.39) mortality relative to the matched reference subjects. Hyperuricemic patients treated with ULT had a lower risk of all-cause death (0.60; 0.41–0.88) relative to hyperuricemic patients who did not receive ULT.

**Conclusion:**

Under-treatment of hyperuricemia has serious negative consequences. Hyperuricemic patients who received ULT had potentially better survival than patients who did not.

## Introduction

Hyperuricemia has recently attracted world-wide attention [1]. Its prevalence has increased to around one-quarter to one-third of the population since the 1980s, which may be related to the growing prevalence of obesity and metabolic syndrome [2]. Hyperuricemia is generally considered asymptomatic. Although symptomatic hyperuricemia with gout attacks and renal lithiasis may be an indication for intervention, an unclear boundary between symptomatic and asymptomatic hyperuricemia is present [3, 4], which may be associated with higher reported risks of all-cause and cardiovascular disease (CVD) mortality in hyperuricemic patients [5–8].

Non-pharmacotherapy with moderately intense physical activity is recommended to reduce the mortality risk that is increased with high serum uric acid (sUA) [9]. However, whether urate lowering therapy (ULT) is beneficial for hyperuricemic patients is still un-determined because of the advanced age and the presence of comorbidities such as chronic kidney disease, CVD, obesity, metabolic syndrome, and alcohol abuse in hyperuricemic patients.

The survival benefit of allopurinol for all-cause death was reported in an observational study of hyperuricemic veterans in the US [10]. A recent cohort study further showed that allopurinol initiation reduces the risk of all-cause mortality by 19% in hyperuricemic patients [11]. A randomized controlled trial in adolescents with early-onset hypertension and hyperuricemia showed that allopurinol reduces systolic and diastolic blood pressure [12]. The benefit of pharmacological intervention for hyperuricemia with allopurinol can be due to inhibition of the renin-angiotensin system, amelioration of hypertension, and lowering of sUA [13, 14].

In our previous demonstration of a beneficial effect of physical activity in patients with hyperuricemia [9], a lower mortality risk in hyperuricemic patients who self-reported ULT use relative to those self-reported no ULT use were noted, when both groups of ULT use and no use were physically active. We hypothesized a potential survival benefit of ULT in patients with hyperuricemia and aimed to extend this research to examine whether use of ULT provides a survival benefit in hyperuricemic patients, especially in those who do not have gout.

## Materials and Methods

### Data source

This retrospective case-matched cohort study used clinical data collected from the nationwide MJ Health Screening Centers, which enroll participants from all regions of Taiwan [9]. All members of the cohort were self-paying participants. The database contained 47,777 individuals who were ≥17 years old and who had a physical checkup in 1996. Individuals were excluded, if they had invalid or missing ULT exposure time (n = 2,351) or clinical measurements (n = 927), if they died before 1997 (n = 52), or if they self-reported use of a ULT at baseline (n = 1,683). The remaining 42,764 individuals were considered for inclusion in the present study. Information on demographics, medical or operational history, and lifestyle factors was collected with a structured questionnaire. Anthropometric measurements and biochemical assays of fasting blood samples were carried out. All participants had provided consent for the release of data. The MJ cohort data have been used in several publications [5, 8].

The MJ dataset is linked to the National Health Insurance Research Database (NHIRD), which records all medical claims for drugs dispensed to Taiwanese patients who are covered by National Health Insurance (NHI), with minimal under-reporting and misclassification [15]. The MJ database is also linked to the National Mortality Registry, which provides the date and cause of all deaths [16]. The Department of Health anonymously performed all record linkages using encrypted data. All traceable personal identifiers were removed from the dataset before statistical analysis to protect patient confidentiality. The Institutional Review Board of the China Medical University Hospital in Taichung (DMR96-IRB-241; DMR99-IRB-074; CMUH103-REC1-020) approved this study.

### Criteria for selecting patients with hyperuricemia and those with gout

Among participants in this MJ cohort, 10,103 subjects with sUA > 7 mg/dL were defined as patients with hyperuricemia. The definition of gout was a patient with a diagnosis code 274.X (of the ninth version of the International Classification of Diseases [ICD-9]) and with concomitant use of a non-steroidal anti-inflammatory drug or colchicine between January 1, 1997 and December 31, 2002 [17]. According to a previous sensitivity analysis of using ICD-code to define gout in the NHIRD [18], subjects who were not gout but were assigned ICD-code of 274.X can include those of simply assessing uric acid level in the NHRI for reimbursement purpose. According to above definition of gout with concomitant medication, we identified 2,646 incident gout patients (1,549 gout patients among hyperuricemic patients; and 1,097 gout patients among 32,661 patients without hyperuricemia) from the examinees of the MJ Health Screening Centers during this period. We excluded all these gout patients from the study (S1 Fig).

### Criteria for selecting patients who received ULT

According to the Taiwanese guideline, asymptomatic hyperuricemic patients with sUA > 9 mg/dL along with comorbidity or patients with sUA > 10 mg/dL are suggested to consult doctors to decide if they should receive ULT [19]. Meanwhile, some Taiwanese physicians follow the Japanese guideline [20] to prescribe ULT in patients with sUA > 8 mg/dL and other complications. Information about ULT that were dispensed from January 1, 1997 to December 31, 2002 was derived from the NHIRD. The most commonly used ULTs in the study cohort were benzbromarone (73.0%), allopurinol (52.6%), probenecid (2.5%), and sulfinpyrazone (0.8%). Of these 8,554 patients with hyperuricemia, 12.1% (n = 1,032) were prescribed ULT. Of the remaining 31,564 reference subjects without hyperuricemia or gout, 1,089 were prescribed a ULT for other reasons, such as renal lithiasis. We excluded these 1,089 subjects from the study. The remaining 30,475 subjects formed the reference cohort (no hyperuricemia, no gout, and no ULT).

The cohort of hyperuricemic patients who did not have gout and were not treated with a ULT (hyperuricemia [HUA] /No gout /No ULT) (n = 7,522), were matched with reference subjects (No HUA /No gout /No ULT) at a 1:3 ratio according to age (within a 5-year age span) and sex. The observation time was calculated from the entry date of the health examination in 1996 to the censored date, which was either the time of death or the end of follow-up (December 31, 2002).

### Outcome and relevant variables

The primary outcome was all-cause mortality, and the secondary outcome was CVD (ICD-9 390–459) mortality. Kaplan-Meier survival curves were used to plot the survival probability of all-cause and CVD mortality for the 30,475 reference subjects (No HUA /No gout /No ULT), the 7,522 hyperuricemic patients who were not treated with ULT (HUA /No gout /No ULT), and the 1,032 hyperuricemic patients who were treated with ULT (HUA /No gout /ULT), all of which (S1 Table) were tested with the log-rank test (S2 Fig).

### Propensity score as the indication probability of ULT

A propensity score (0 to 1) was used to determine the likelihood of a hyperuricemic patient being prescribed a ULT by a physician given his or her individual characteristics [21]. This analysis was performed separately for all ULT-treated patients and for those treated with allopurinol only or benzbromarone only. Stepwise logistic regression analysis was conducted to determine significant predictors for ULT use from 104 baseline variables defined at the physical check-up in 1996, including demographics, comorbidities, anthropometric measurements, and medication (C-statistic = 0.88). Patients with hyperuricemia treated with ULT and those not treated with ULT were matched at a 1:1 ratio based on the propensity score according to a greedy algorithm and the index date of ULT prescription [22]. The index dates for reference subjects not treated with ULT were randomly assigned and then used to match each reference subject with a hyperuricemic patient based on his/her date of ULT prescription. We matched one reference subject for each identified hyperuricemic patient treated with ULT. Matching for propensity score was performed to attenuate the bias associated with the drug indication, and matching for the index date of ULT prescription was performed to reduce the immortal time bias between treatment and no treatment. Of these hyperuricemic patients treated with ULT, 1,024 patients (99.2% completeness of 1,032 patients) were successfully matched to a non-ULT hyperuricemic patient with the nearest propensity score by applying a caliper of 0.05 on the propensity score scale and the same index date of ULT prescription [23]. The observation time was calculated from the index date of ULT prescription until the time of death or the censored date. Using a similar matching technique in a separate analysis of the same group of participants, we derived 273 matched pairs of allopurinol-only users and 584 matched pairs of benzbromarone-only users among hyperuricemic patients. Each identified hyperuricemic patient treated with allopurinol only or benzbromarone only was matched with one patient who did not receive ULT (HUA /No gout /No ULT).

### Statistical analysis

Baseline demographic data were compared between hyperuricemic patients and their respective reference groups using the Student’s *t*-test for continuous data and the chi-square test for categorical data. The mortality events resulting from all causes and CVD were compared (1) between hyperuricemia patients who did not receive ULT and the 1:3 matched reference subjects (No HUA /No gout /No ULT), and (2) between the 1:1 matched pairs of hyperuricemic patients with and without ULT. The Cox proportional hazard model was used to estimate the adjusted hazard ratio (HR) of all-cause and CVD mortality.

#### Subgroup analysis for ULT use in hyperuricemic patients

The concomitant use of anti-hypertensive [24], anti-diabetic [25], and lipid-lowering drugs [26] may lower sUA levels [27]. The effect of ULT and concomitant medication on mortality in hyperuricemic patients was analyzed by stratifying the patients with respect to the presence or absence of each concomitant medication. The hyperuricemic patients with ULT were compared with their 1:1 matched counterparts within each stratified subgroup. A similar matching method with a 1:1 ratio was applied by stratifying hyperuricemic patients with ULT with respect to the cumulative duration of ULT use. This approach maintains comparability between the case and reference cohorts in terms of the baseline characteristics [17]. All analyses were performed using SAS statistical software version 9.3 (SAS Institute, Cary, NC).

## Results

### Demographic data

In total, 39,029 subjects satisfied the inclusion criteria and were followed up for a mean of 6.4 years. There were 3,088 hyperuricemic patients who did not receive ULT (HUA /No gout /No ULT) were matched with 9,264 reference subjects (No HUA /No gout /No ULT) (i.e., one hyperuricemic patient was matched with three corresponding reference subjects). Table 1 shows the demographic, lifestyle, and clinical characteristics of the two subgroups of patients after matching with their paired controls. Some of the baseline characteristics differed. The sUA level was 7.7 mg/dL in hyperuricemic patients who did not receive ULT and 5.5 mg/dL in reference subjects. Hyperuricemic patients who did not receive ULT, had a worse metabolic profile of systolic blood pressure, triglyceride, and body mass index and a higher prevalence of comorbidities of hypertension, heart disease and diabetes mellitus than the reference subjects. Between hyperuricemic patients who received ULT (HUA /No gout /ULT) and the reference group of hyperuricemic patients who did not receive ULT (HUA /No gout /No ULT), who were matched by propensity score and index date, a similar sUA level (8.2 mg/dL, *p* = 0.76; Table 1) was noted. Balanced and similar demographic data were also available for the matched pairs of hyperuricemic patients who did and did not receive ULT.

**Table 1:** Baseline demographic, lifestyle, and clinical characteristics of hyperuricemic patients and the matched reference subjects.

Characteristics	HUA (+),ULT (−)n = 3,088	HUA (−),ULT (−)n = 9,264	*p*	HUA (+),ULT (+)n = 1,024	HUA (+),ULT (−)n = 1,024	*p*
Age, years	43.3 ± 15.2	42.8 ± 15.1	0.12	51.6 ± 14.8	51.5 ± 14.7	0.93
Male, n (%)	1689 (54.7)	5253 (56.7)	0.06	731 (71.4)	725 (70.8)	<0.01
Follow-up time, years	6.4 ± 0.7	6.5 ± 0.6	0.01	6.4 ± 0.8	6.3 ± 0.9	0.01
sUA, mg/dL	7.7 ± 0.7	5.5 ± 0.9	<0.01	8.2 ± 1.0	8.2 ± 1.0	0.76
SBP, mmHg	122.9 ± 20.3	121.6 ± 20.6	<0.01	134.7 ± 22.8	134.0 ± 22.5	0.51
Cholesterol, mg/dL	194.5 ± 37.8	193.23 ± 37.7	0.12	207.7 ± 40.1	208.8 ± 37.9	0.54
HDL-C, mg/dL	45.3 ± 12.9	45.7 ± 13.1	0.22	43.8 ± 13.7	43.3 ± 13.5	0.43
Triglyceride, mg/dL	111.6 ± 56.6	107.0 ± 56.8	<0.01	157.9 ± 86.8	154.9 ± 94.1	0.45
Glucose, mg/dL	98.7 ± 19.9	98.3 ± 19.3	0.33	103.4 ± 23.2	102.6 ± 19.3	0.42
eGFR, mL/min per 1.73 m^2^	80.7 ± 15.9	80.9 ± 14.2	0.72	71.1 ± 16.8	71.4 ± 15.1	0.68
BMI, kg/m^2^	23.4 ± 3.5	23.2 ± 3.3	<0.01	25.6 ± 3.4	25.5 ± 3.6	0.60
**Comorbidity**						
Hypertension, n (%)	53 (1.7)	116 (1.3)	0.03	27 (2.6)	27 (2.6)	0.84
Heart disease, n (%)	251 (8.1)	641 (6.9)	0.05	243 (23.7)	239 (23.3)	1.00
Diabetes mellitus, n (%)	14 (0.5)	33 (0.4)	0.05	10 (1.0)	10 (1.0)	0.77
**Alcohol consumption**			0.45			0.67
Never, n (%)	352 (11.4)	1073 (11.6)		115 (11.2)	123 (12.0)	
Abstained, n (%)	1478 (47.9)	4484 (48.4)		429 (41.9)	413 (40.3)	
1–2 drinks/week, n (%)	93 (3.0)	309 (3.3)		51 (5.0)	39 (3.8)	
3–4 drinks/week, n (%)	818 (26.5)	2421 (26.1)		261 (25.5)	292 (28.5)	
Daily, n (%)	256 (8.3)	748 (8.1)		119 (11.6)	110 (10.7)	
Missing data, n (%)	91 (3.0)	229 (2.5)		49 (4.8)	47 (4.6)	
**Cigarette smoking**			0.74			0.97
Never, n (%)	446 (14.4)	1331 (14.4)		139 (13.6)	141 (13.8)	
Abstained, n (%)	1453 (47.1)	4431 (47.8)		411 (40.1)	414 (40.4)	
Occasionally, n (%)	191 (6.2)	544 (5.9)		127 (12.4)	125 (12.2)	
Often, n (%)	130 (4.2)	359 (3.9)		40 (3.9)	45 (4.4)	
Daily, n (%)	278 (9.0)	777 (8.4)		97 (9.5)	87 (8.5)	
Missing data, n (%)	590 (19.1)	1822 (19.7)		210 (20.5)	212 (20.7)	
**Smoking amount**			0.67			0.51
None or missing, n (%)	2040 (66.1)	6063(65.5)		587(57.3)	601(58.7)	
<5 cigarettes per day, n (%)	164 (5.3)	466 (5.0)		60 (5.9)	53 (5.2)	
5–10 cigarettes per day, n (%)	197 (6.4)	631 (6.8)		74 (7.2)	72 (7.0)	
11–19 cigarettes per day, n (%)	476 (15.4)	1489 (16.1)		203 (19.8)	196 (19.1)	
1–2 packs per day, n (%)	208 (6.7)	605 (6.5)		98 (9.6)	101 (9.9)	
>2 packs per day, n (%)	3 (0.1)	10 (0.1)		2 (0.2)	2 (0.1)	

Regarding alcohol consumption and cigarette smoking, “Never” means that the subjects have never consumed alcohol or smoked and “Abstained” indicates that although the subjects previously consumed alcohol or smoked, they have quit.

Abbreviations: sUA: serum uric acid; SBP: systolic blood pressure; BMI: body mass index; HDL-C: high-density lipoprotein-cholesterol; eGFR: estimated glomerular filtration rate by using Modification of Diet in Renal Disease (MDRD) formula.

HUA (+), ULT (–): hyperuricemic patients who did not have gout and did not receive ULT (n = 3,088)

HUA (–), ULT (–): non-hyperuricemic, no gout and non-ULT reference subjects who were matched 1:3 to the subgroup of HUA (+), ULT (–) by age and gender (n = 9,264)

HUA (+), ULT (+) versus HUA (+), ULT (–): 1,024 pairs of hyperuricemic patients who did not have gout and who were treated with or without ULT, matched by propensity score and the index date of ULT prescription.

### Kaplan-Meier survival curve

**Fig 1:**
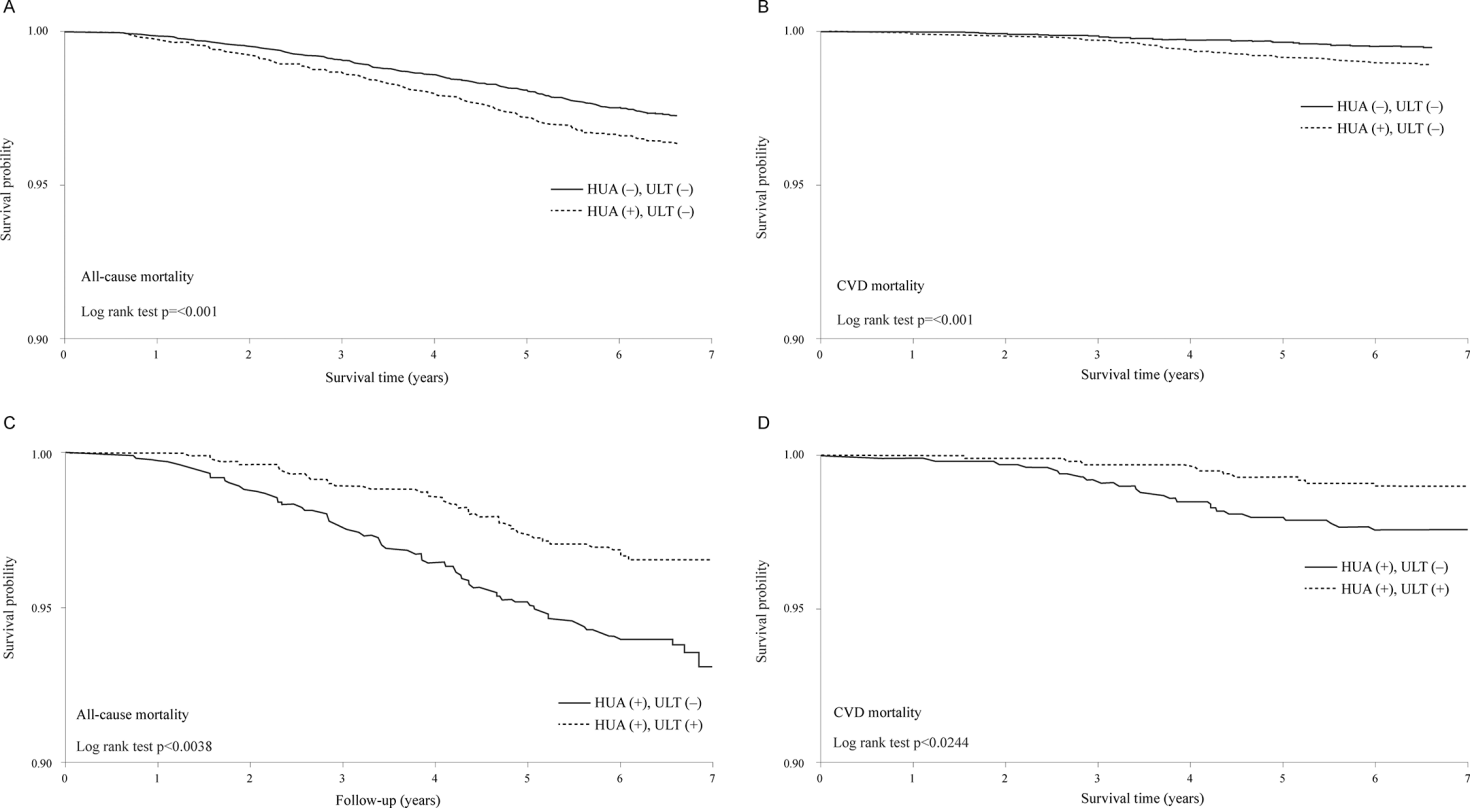
Survival curves comparing the 3,088 untreated hyperuricemic patients (HUA /No Gout /No ULT) and 9,264 matched reference individuals (No HUA /No Gout /No ULT) (Figs 1A and 1B), and comparing the 1,024 matched pairs of hyperuricemic patients who did and did not receive ULT (Figs 1C and 1D).

Fig 1 shows the survival curves of all-cause and CVD mortality for the untreated hyperuricemic patients (HUA /No gout /No ULT) and the matched reference subjects (No HUA /No gout /No ULT) and also compares the survival curves between hyperuricemic patients who used ULT and those who did not. Overall, the untreated hyperuricemic patients had poorer outcomes than the matched reference subjects (*p* < 0.001) (Fig 1A and 1B), and among the hyperuricemic patients, the ULT-users (HUA /No gout /ULT) had better outcomes than the matched non-users (HUA /No gout /No ULT) (*p* < 0.05) (Fig 1C and 1D).

### Mortality risks associated with untreated hyperuricemia

Matching was performed to improve the similarity of confounding variables across comparison groups. Comparison of hyperuricemic patients who did not use ULT (HUA /No gout /No ULT), to the matched reference group (No HUA /No gout /No ULT) showed a marginally significant effect on all-cause mortality (adjusted HR 1.24; 95% confidence interval (CI) 0.97–1.59) due to attrition of case number after matching procedure, and a significant risk of hyperuricemia for CVD mortality (adjusted HR 2.13; 95% CI 1.34–3.39) (Table 2).

**Table 2:** Mortality risk based on the presence of hyperuricemia and ULT.

				All-cause mortality					CVD mortality				
HUA	ULT	N	PY	E	M	MRR	HR (95% CI)	*p*	E	M	MRR	HR (95% CI)	*p*
–^a^	–	9264	59976.73	212	3.53		Reference		43	0.72		Reference	
+^a^	–	3088	19883.32	90	4.53	1.28	1.24 (0.97–1.59)	0.08	31	1.56	2.17*	2.13 (1.34–3.39)	<0.01
+^b^	–	1024	6465.26	71	10.98		Reference		22	3.4		Reference	
+^b^	+	1024	6565.42	45	6.85	0.62*	0.60 (0.41–0.88)	0.01	15	2.28	0.67	0.63 (0.32–1.22)	0.17

^a^HRs were adjusted for age, gender, lifestyle (smoking, drinking, and exercise), and comorbidity (obesity, hypertension, diabetes, renal failure, and hepatitis).

^b^HRs were adjusted for the propensity score.

*p < 0.05

Abbreviations: CVD: cardiovascular disease; HUA: hyperuricemia; ULT: urate-lowering therapy; N: number; PY: person-years; E: event; M: mortality rate; MRR: mortality rate ratio; HR: hazard ratio; 95% CI: 95% confidence interval

### ULT in hyperuricemic patient

In the 1:1 matched groups of hyperuricemic patients who did and did not receive ULT, the all-cause and CVD mortality rates of ULT users were lower than the rates in non-ULT users (Table 2). Hyperuricemic patients who receive ULT had significantly lower mortality risk from all causes (adjusted HR 0.60; 95% CI 0.41–0.88) relative to the matched hyperuricemic patients who did not use ULT. However, the comparison of the CVD mortality risk between ULT users and non-users was not statistically significant (HR 0.63; 95% CI 0.32–1.22) due to small number of mortality events.

### Allopurinol or benzbromarone use in hyperuricemic patients

A potential effect of the type of ULT on mortality outcomes was examined. We compared all-cause and CVD mortalities in hyperuricemic patients who used allopurinol (a xanthine oxidase inhibitor) or benzbromarone (a uricosuric agent) with those who did not use any ULT (S2 Table). Although the mortality rates were generally lower in the ULT users than the reference patients of non-users, a significantly lower risk of death was found solely in patients who used benzbromarone relative to the corresponding matched non-ULT users (adjusted HR 0.58; 95% CI 0.34–0.99). We note, however, that the small number of mortality events makes these risk estimates somewhat unreliable (Table 3).

**Table 3:** Mortality risk based on the presence of hyperuricemia and ULT with either allopurinol or benzbromarone.

				All-cause mortality					CVD mortality				
HUA^a^	ULT	N	PY	E	M	MRR	HR (95% CI)	*p*	E	M	MRR	HR (95% CI)	*p*
+	–	273	1719.39	19	11.05		Reference		6	3.49		Reference	
+	A	273	1728.23	17	9.84	0.89	1.00 (0.51–1.95)	1.00	3	1.74	0.50	0.49 (0.12–2.00)	0.32
+	–	584	3701.79	37	10.00		Reference		14	3.78		Reference	
+	B	584	3750.27	23	6.13	0.61	0.58 (0.34–0.99)	0.05	11	2.93	0.78	0.70 (0.31–1.56)	0.38

^a^HRs were adjusted for the propensity score.

Abbreviations: CVD: cardiovascular disease; HUA: hyperuricemia; ULT: urate-lowering therapy; N: number; PY: person-years; E: event; M: mortality rate; MRR: mortality rate ratio; HR: hazard ratio; 95% CI: 95% confidence interval; A: allopurinol; B: benzbromarone

### Effect of ULT and concomitant medication

**Fig 2:**
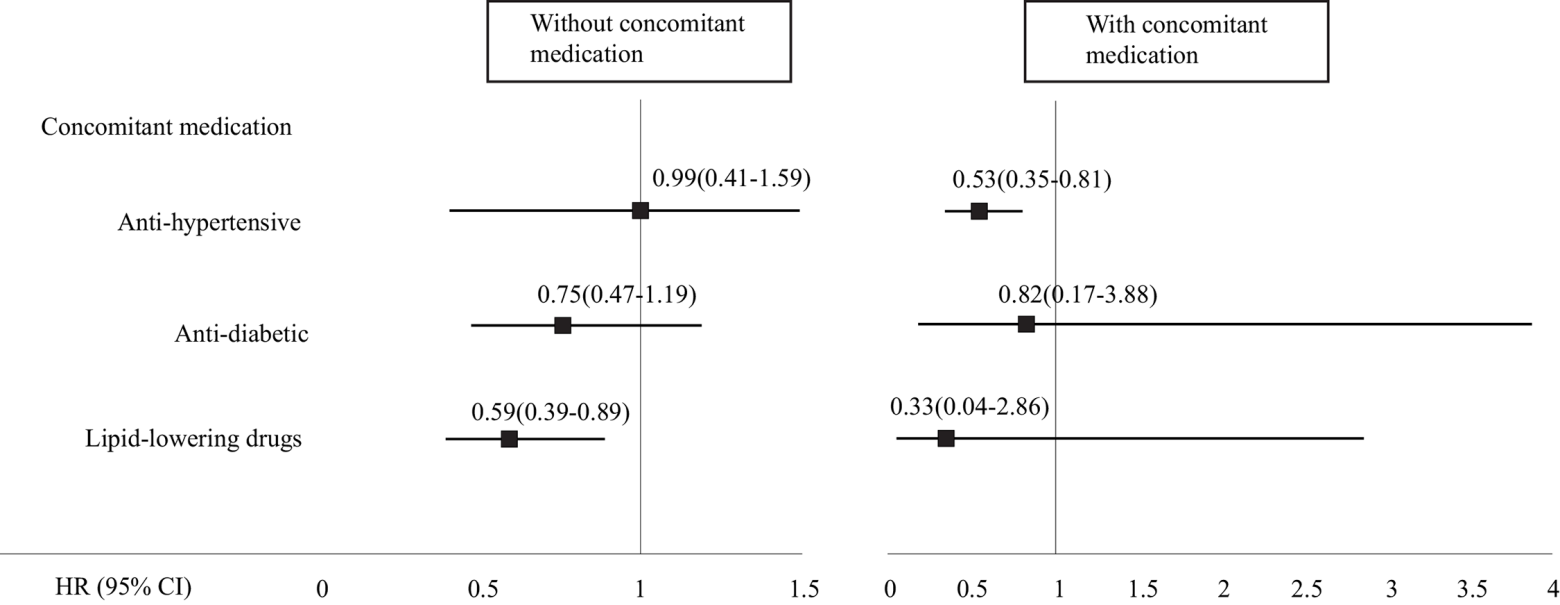
Mortality risk (HR [95% CI]) of hyperuricemia patients according to the use of ULT in subgroups stratified by the presence or absence of concomitant medications including anti-hypertensive, anti-diabetic, and lipid-lowering drugs.

In each subgroup stratified according to concomitant medication, which consisted of anti-hypertensive, anti-diabetic, and lipid-lowering agents, fewer mortality events were generally seen in hyperuricemic patients who used ULT than in the 1:1 matched non-users. However, only subgroups of patients with hyperuricemia in which we considered concomitant use of anti-hypertensive medication (721 pairs) and no lipid-lowering medication (860 pairs) had sufficient power for a risk estimate of all-cause mortality (Fig 2 and S3 Table). We observed an independent effect of ULT on all-cause mortality in hyperuricemic patients who did not use lipid-lowering agents. In the presence of concomitant anti-hypertensive drugs, ULT reduced the risk of all-cause mortality by 47%.

### Duration of ULT use

**Fig 3:**
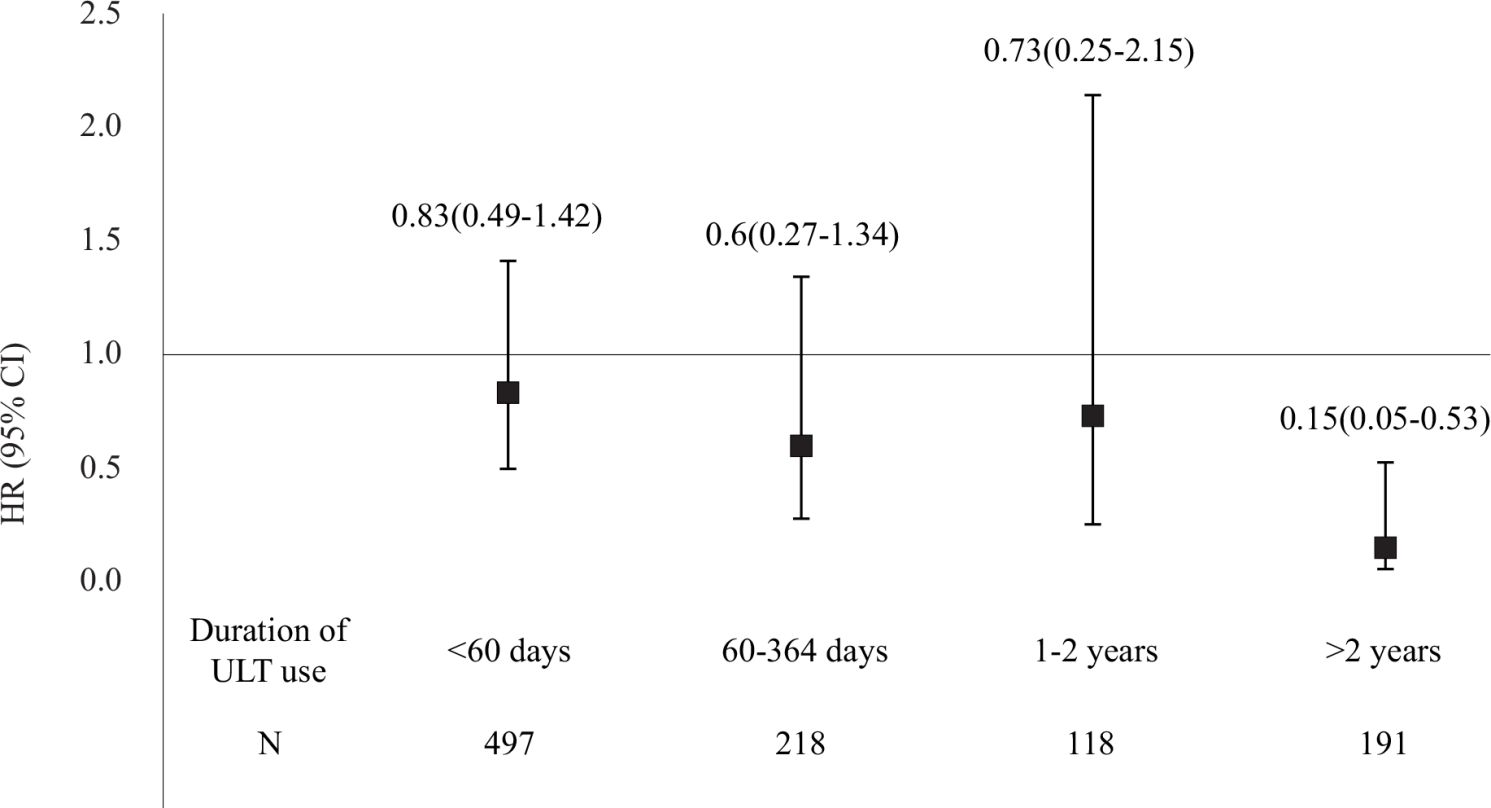
Mortality risk stratified according to the duration of ULT use.

Among the hyperuricemic patients who received ULT, 48.5% of these patients used ULT cumulatively for <60 days (Fig 3 and S3 Table). We observed no significant differences in mortality rates between hyperuricemic patients who used ULT for ≤2 years and the 1:1 matched non-ULT users. The all-cause mortality rate of hyperuricemic patients who used ULT for >2 years was significantly lower than that of the 1:1 matched non-ULT users. However, too few CVD events were present to demonstrate a possible impact of ULT on CVD mortality.

## Discussion

Previous studies indicated an independent association between hyperuricemia and increased risk of premature all-cause and CVD death [5, 8]. Our findings in this retrospective cohort study confirm and extend these reports of a risk due to hyperuricemia and address an enhanced and significant CVD mortality risk in hyperuricemic patients (HUA /No gout /No ULT) relative to reference subjects (No HUA /No gout /No ULT). A potentially positive correlation of the survival benefit between ULT use and mortality was demonstrated in patients with hyperuricemia who were matched with non-users according to their propensity score and the index date of ULT prescription.

Throughout the follow-up period, compared with the matched non-ULT users, ULT users had lower mortality rates, and a significant survival benefit of ULT use was demonstrated after >2 years of use. The observation for each matched pair with variably cumulative ULT use intervals started from the same index date. This finding, based on the comparison between ULT users and their 1:1 matched non-ULT users, is consistent with a recent general population study indicating an association between reduced risk of death in patients with hyperuricemia and initiation of treatment with allopurinol [11].

Our present study did not, however, show a beneficial effect of allopurinol on premature mortality that was due to hyperuricemia. This negative finding is consistent with a recent Taiwanese report using the database from NHIRD [28]. One contributing factor to this finding is that our current study showed far fewer allopurinol users than benzbromarone users. This may be due to a potential hypersensitivity risk of mild cutaneous reaction and serious hypersensitivity reactions, including Steven-Johnson syndrome with allopurinol, which was the number one insurance compensation and medical hazard reported in 1999–2011 in Taiwan [29]. A reported risk association between the HLA-B*5801 allele and allopurinol hypersensitivity syndrome in Asians [30, 31] may at least partially explain this catastrophic adverse effect. In contrast to the lack of an effect of allopurinol, a significant risk reduction in all-cause mortality was demonstrated in hyperuricemic patients treated with benzbromarone only. A better tolerability for benzbromarone in Taiwanese patients is suspected, as indicated by a higher frequency of prescriptions for benzbromarone in Taiwan [32]. However, the fact that benzbromarone was once withdrawn from the market because of serious hepatotoxicity is noteworthy [33]. Although a possible adverse effect on CVD events was reported for febuxostat, a new selective xanthine-oxidase inhibitor [34], re-evaluation of its potential impact on mortality will be interesting in the future.

Although uric acid is regarded as an anti-oxidant, it can act as a damage-associated molecular pattern that contributes to gout and its comorbidities of atherosclerosis and metabolic syndrome [35]. The role of uric acid may contribute to activate oxidative stress, reduce nitric oxide production and increase reactive oxygen species (ROS) in blood vessels [36], which leads to aggravate vascular inflammation, inhibit endothelial-cell growth and result in proliferation of vascular smooth muscle cells. Multiple organs including kidney may be systemically involved due to endothelial dysfunction [37] with potentiation of arthrosclerosis and thrombogenesis [14]. Drugs that lower the sUA level may suppress inflammatory responses that result from the NLRP3 inflammasome [38]. The ability of allopurinol or benziodarone to lower both sUA levels and blood pressure has been demonstrated in an animal study [14]. In an experimental model of hyperuricemic rats, an early state of activated renin-angiotensin system and reduced circulating nitric oxide can be reversed to a state of normal blood pressure by ULT [13]. Rabbits pre-treated with ULT show reduced levels of uric acid and xanthine after arterial occlusion, as well as less severe early and late cerebral injury, as compared with untreated rabbits [39]. Our observations that ULT can reduce mortality in hyperuricemic patients are consistent with these reports.

Our study has several limitations. First, this was not a randomized clinical trial, so we cannot attribute all the observed mortality risk or benefit exclusively to ULT. Nonetheless, in the model we used, we adjusted for several potential variables that may affect mortality. A balanced pattern of known comorbidities (e.g., hypertension, diabetes, and obesity) between ULT users and non-users in hyperuricemic patients was established by the greedy matching technique. ULT appeared to have a direct and independent effect on all-cause mortality in Taiwanese patients with hyperuricemia who did not have gout. Second, our information on ULT prescriptions came from the NHI database, a record of dispensed drug claims. Compliance with ULT may have been poor in many hyperuricemic patients [40], making evaluation of the effect of ULT on outcomes difficult. However, use of a rigorous matching process to maximize the statistical power and a reasonably long observation period to assess the cumulative drug effects on outcomes may have compensated for these non-differential biases. Third, a limitation of our study is that thiazide diuretics which can induce hyperuricemia, are often prescribed with other anti-hypertensive. It was not easy to separate thiazide diuretics from other anti-hypertensive in the stratified analysis. Nevertheless, a survival benefit was found when ULT is used concomitantly with anti-hypertensive for its effect in reducing arterial pressure and increasing survival. Fourth, because of a regulation change in Taiwan, our study had a limitation in connecting database to NHIRD after the year of 2002. We can, thus, only focus on ULT of allopurinol and benzbromarone without extending to the new ULT of febuxostat, which did not appear in the Taiwanese market until 2013. Fifth, one may argue that the risk reduction was due to the effect of ULT on lowering sUA and/or blood pressure [41]; this is a limitation of our current cohort, which included very few repeated measurements of sUA levels. However, by relying on a single measurement, this study was able to more simply assess the relationship between hyperuricemia and mortality risks. Lastly, although members of the cohort were self-paying participants of an above-average socioeconomic class, the HRs were internally compared, so they would not have been affected by the influence of socioeconomic class.

Our study also has several strengths. A potential bias, which can result from different baseline risks between hyperuricemic patients who were or were not treated with ULT was attenuated by propensity score matching. The baseline characteristics between ULT users and non-users were balanced and showed no significant difference. In addition, a potential bias caused by the immortal time effect was also reduced, because if non-ULT hyperuricemic users died before the date of initial ULT prescription to their matched ULT users, we reselected subjects by matching the index date of ULT prescription. In addition, the current study relied on the large amount of recorded information that is available in the national database to examine the potential benefit of ULT in hyperuricemic patients. The use of a large number of measured covariates (104 in total) may overcome the limitation of using propensity scores in 1:1 matched case-cohort analyses [21]. The model generated with the stepwise selection method had a good C-statistic (0.88), and almost 99.2% of treated hyperuricemic patients were included. Moreover, the national census and accuracy of national death files in Taiwan are known to be complete [16] and no reason exists to suspect the presence of a non-differential bias.

We recently reported serious negative consequences on mortality in under-treated gout patients, and gout patients who received ULT had significantly better survival than those who did not [17]. This current population study that focused on hyperuricemic patients who did not have gout suggests that untreated hyperuricemic patients also suffer negative consequences, especially on CVD mortality. ULT, especially benzbromarone alone, significantly improved survival outcomes in hyperuricemic patients relative to untreated patients. Allopurinol use in the Asian population should be considered for a potential risk because of the HLA-B*5801 allele polymorphism [29–31]. To generalize our data to other ethnic populations, large controlled clinical trials should be performed in the future to definitively resolve this issue.

## Supporting Information

S1 Fig Flow diagram of study design: matched case-cohort study. (PDF)

S2 Fig Survival curves of hyperuricemic patients and the reference non-hyperuricemic, non-ULT individuals. (PDF)

S1 Table Demographic, lifestyle, and clinical characteristics of the studied subjects (N = 39,029). (DOCX)

S2 Table Demographics of hyperuricemic patients with allopurinol-only use or benzbromarone-only use and their matched counterparts. (DOCX)

S3 Table Mortality risk according to the presence of ULT use in subgroups stratified by concomitant medications and by duration of ULT use. (DOCX)

S1 Text Data Availability Statement. (DOCX)
