# Supplementary material for: Effect of Urate-Lowering Therapy on All-Cause and Cardiovascular Mortality in Hyperuricemic Patients without Gout: A Case-Matched Cohort Study
Source: PLoS One. 2015 Dec 18;10(12):e0145193. doi: 10.1371/journal.pone.0145193 (PMC4684295; doi:10.1371/journal.pone.0145193)

S1 Fig. Flow diagram of study design: matched case-cohort study

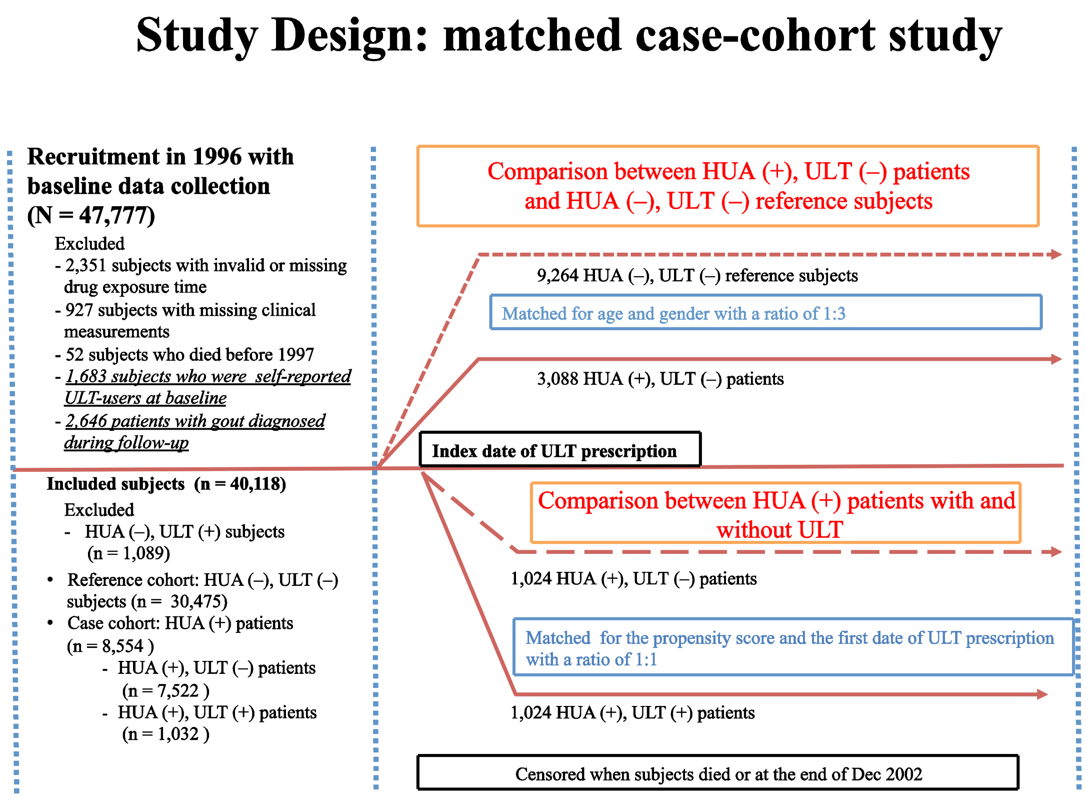

Supplement: S1 Fig — (PDF) [file pone.0145193.s001.pdf]
