# Supplementary material for: Effect of Urate-Lowering Therapy on All-Cause and Cardiovascular Mortality in Hyperuricemic Patients without Gout: A Case-Matched Cohort Study
Source: PLoS One. 2015 Dec 18;10(12):e0145193. doi: 10.1371/journal.pone.0145193 (PMC4684295; doi:10.1371/journal.pone.0145193)
Supplement: S2 Fig — (PDF) [file pone.0145193.s002.pdf]

S2 Fig. Survival curves of hyperuricemic patients and the reference non-hyperuricemic, non-ULT individuals.

(A)

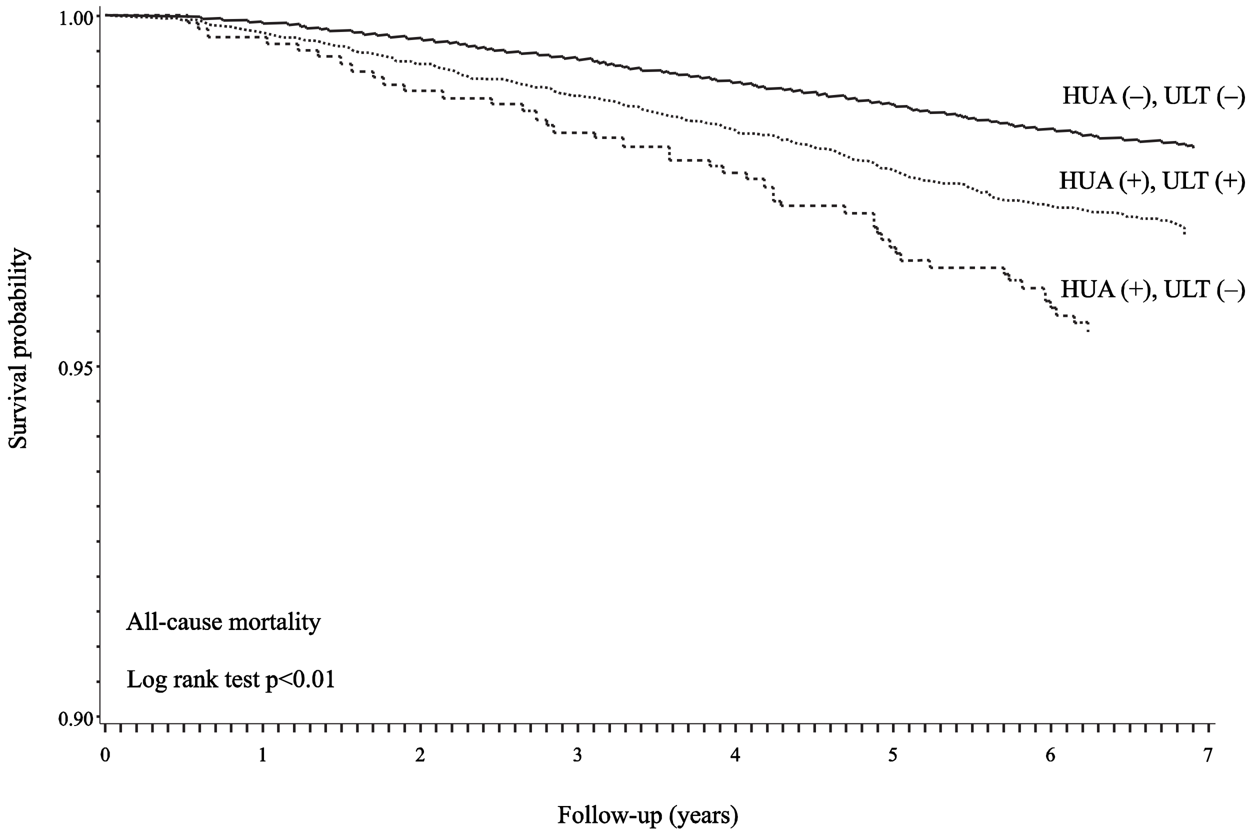

(B)

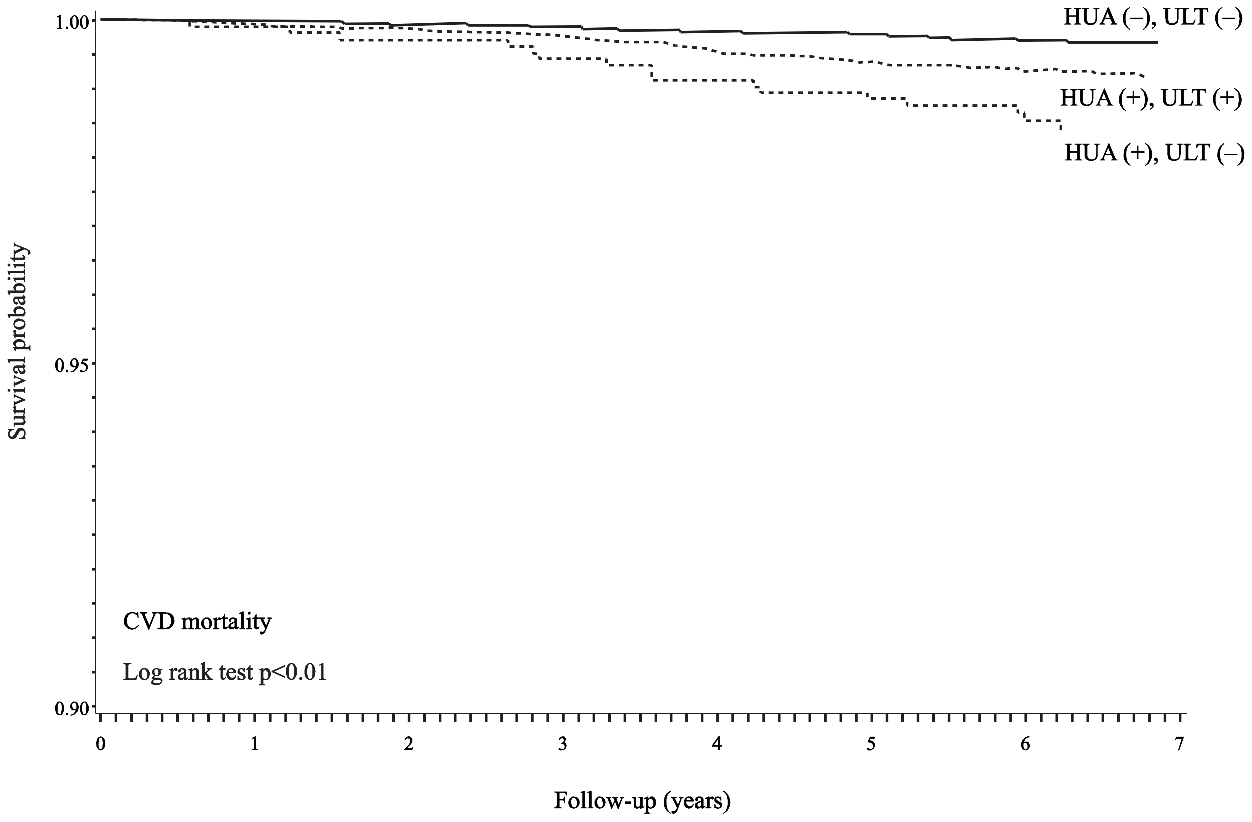

Data for all-cause mortality and CVD mortality are shown for the entire pool of patients (i.e., as shown in Supplementary Table in S1 Table).

Abbreviations: HUA (−), ULT (−): 30,475 non-hyperuricemic, non-gout, non-ULT users;

HUA (+), ULT (−): 7,522 hyperuricemic patients who did not receive ULT;

HUA (+), ULT (+): 1,032 hyperuricemic patients who received ULT
