# Supplementary material for: Effect of Urate-Lowering Therapy on All-Cause and Cardiovascular Mortality in Hyperuricemic Patients without Gout: A Case-Matched Cohort Study
Source: PLoS One. 2015 Dec 18;10(12):e0145193. doi: 10.1371/journal.pone.0145193 (PMC4684295; doi:10.1371/journal.pone.0145193)
Supplement: S1 Table — (DOCX) [file pone.0145193.s003.docx]

**S1 Table.** Demographic, lifestyle, and clinical characteristics of the studied subjects (N = 39,029)

| Characteristic | **HUA (−),**  **ULT (−)**  **n = 30,475** | **HUA (+),**  **ULT (−)**  **n = 7,522** | **HUA (+),**  **ULT (+)**  **n = 1,032** | ***p*** |
| --- | --- | --- | --- | --- |
| Age, years | 40.7 ± 14.2 | 51.7 ± 14.8 | 40.4 ± 14.5 | <0.001 |
| Male, n (%) | 10287 (33.8) | 736 (71.3) | 5964 (79.3) | <0.001 |
| Follow-up time, years | 6.5 ± 0.5 | 6.4 ± 0.8 | 6.4 ± 0.6 | <0.001 |
| sUA, mg/dL | 5.2 ± 1.0 | 8.2 ± 1.0 | 7.9 ± 0.8 | <0.001 |
| SBP, mmHg | 118.3 ± 20.0 | 134.9 ± 22.9 | 123.8 ± 19 | <0.001 |
| Cholesterol, mg/dL | 191.1 ± 36.9 | 207.8 ± 40.2 | 198.8 ± 37.9 | <0.001 |
| HDL-C, mg/dL | 47.9 ± 13.6 | 43.8 ± 13.7 | 42.6 ± 13.0 | <0.001 |
| Triglyceride, mg/dL | 98.8 ± 58.5 | 158.2 ± 86.8 | 138.1 ± 84.8 | <0.001 |
| Glucose, mg/dL | 97.9 ± 23.4 | 103.5 ± 23.3 | 98.2 ± 15.7 | <0.001 |
| eGFR, mL/min per 1.73 m^2^ | 84.4 ± 15.6 | 70.9 ± 16.8 | 78.6 ± 14.6 | <0.001 |
| BMI, kg/m^2^ | 22.6 ± 3.3 | 25.6 ± 3.4 | 24.5 ± 3.5 | <0.001 |
| **Comorbidities** |  |  |  |  |
| Hypertension, n (%) | 345 (1.1) | 27 (2.6) | 84 (1.1) | <0.001 |
| Heart disease, n (%) | 1597 (5.2) | 250 (24.2) | 579 (7.7) | <0.001 |
| Diabetes mellitus, n (%) | 122 (0.4) | 10 (1.0) | 23 (0.3) | 0.0063 |
| **Alcohol consumption** |  |  |  | <0.001 |
| Never, n (%) | 3969 (13.0) | 117 (11.3) | 675 (9.0) |  |
| Abstained, n (%) | 16106 (52.8) | 433 (42.0) | 3106 (41.3) |  |
| 1–2 drinks/week, n (%) | 676 (2.2) | 52 (5.0) | 264 (3.5) |  |
| 3–4 drinks/week, n (%) | 6760 (22.2) | 262 (25.4) | 2451 (32.6) |  |
| Daily, n (%) | 2430 (8.0) | 119 (11.5) | 800 (10.6) |  |
| Missing data, n (%) | 534 (1.8) | 49 (4.7) | 226 (3.0) |  |
| **Cigarette smoking** |  |  |  | <0.001 |
| Never, n (%) | 5088 (16.7) | 142 (13.8) | 837 (11.1) |  |
| Abstained, n (%) | 16065 (52.7) | 414 (40.1) | 3192 (42.4) |  |
| Occasionally, n (%) | 1248 (4.1) | 128 (12.4) | 644 (8.6) |  |
| Often, n (%) | 980 (3.2) | 40 (3.9) | 369 (4.9) |  |
| Daily, n (%) | 3062 (10.0) | 97 (9.4) | 640 (8.5) |  |
| Missing data, n (%) | 4032 (13.2) | 211 (20.4) | 1840 (24.5) |  |
| **Smoking amount** |  |  |  | <0.001 |
| None or missing, n (%) | 23238 (76.3) | 592 (57.4) | 4212 (56) |  |
| <5 cigarettes per day, n (%) | 1321 (4.3) | 61 (5.9) | 470 (6.2) |  |
| 5–10 cigarettes per day, n (%) | 1469 (4.8) | 74 (7.2) | 576 (7.7) |  |
| 11–19 cigarettes per day, n (%) | 3135 (10.3) | 204 (19.8) | 1564 (20.8) |  |
| 1–2 packs per day, n (%) | 1297 (4.3) | 99 (9.6) | 695 (9.2) |  |
| >2 packs per day, n (%) | 15 (0.0) | 2 (0.2) | 5 (0.1) |  |

Baseline demographic data of the three groups are compared using the One-way ANOVA test for continuous data and the chi-square test for categorical data. Regarding alcohol consumption and cigarette smoking, “Never” indicates that the subjects never consumed alcohol or smoked, and “Abstained” indicates that although the subjects previously consumed alcohol or smoked, they have quit.

Abbreviations: sUA: serum uric acid; SBP: systolic blood pressure; BMI: body mass index; HDL-C: high density lipoprotein-cholesterol; eGFR: estimated glomerular filtration rate.
