# Supplementary material for: Effect of Urate-Lowering Therapy on All-Cause and Cardiovascular Mortality in Hyperuricemic Patients without Gout: A Case-Matched Cohort Study
Source: PLoS One. 2015 Dec 18;10(12):e0145193. doi: 10.1371/journal.pone.0145193 (PMC4684295; doi:10.1371/journal.pone.0145193)
Supplement: S2 Table — (DOCX) [file pone.0145193.s004.docx]

**S2 Table.** Demographics of hyperuricemic patients with allopurinol-only use or benzbromarone-only use and their matched counterparts

| **Characteristics** | **HUA (+),**  **Allopurinol (+)** | **HUA (+),**  **ULT (−)** | ***p*** | **HUA (+),**  **Benzbromarone (+)** | **HUA (+),**  **ULT (−)** | ***p*** |
| --- | --- | --- | --- | --- | --- | --- |
| Age, years | 51.1 ± 15.2 | 51.1 ± 14.8 | 0.98 | 51 ± 14.5 | 51.3 ± 15 | 0.69 |
| Male, n (%) | 192 (70.3) | 211 (77.3) | <0.01 | 412 (70.5) | 398 (68.2) | <0.01 |
| Follow-up time, years | 6.3 ± 1.0 | 6.3 ± 0.9 | 0.68 | 6.4 ± 0.7 | 6.3 ± 0.9 | 0.07 |
| sUA, mg/dL | 8.1 ± 1.0 | 8.2 ± 1.0 | 0.86 | 8.1 ± 0.9 | 8.1 ± 1.0 | 0.80 |
| SBP, mmHg | 132.7 ± 22.8 | 130.9 ± 22.1 | 0.37 | 134.6 ± 22.5 | 134.4 ± 22.6 | 0.84 |
| Cholesterol, mg/dL | 206.0 ± 43.0 | 210.4 ± 38.5 | 0.21 | 208.5 ± 39.4 | 207.6 ± 37.4 | 0.68 |
| HDL-C, mg/dL | 43.8 ± 13.5 | 43.6 ± 14.1 | 0.87 | 43.9 ± 14.0 | 43.0 ± 13.3 | 0.27 |
| Triglyceride, mg/dL | 149.0 ± 89.5 | 153.5 ± 107.8 | 0.59 | 160.5 ± 85.8 | 154.3 ± 90.5 | 0.23 |
| Glucose, mg/dL | 102.8 ± 21.7 | 102.3 ± 19.2 | 0.77 | 103.6 ± 24.3 | 101.9 ± 17.4 | 0.18 |
| eGFR, mL/min per 1.73 m^2^ | 69.9 ± 18.1 | 71.7 ± 14.7 | 0.20 | 72.2 ± 16.1 | 71.7 ± 15.2 | 0.59 |
| BMI, kg/m^2^ | 25.4 ± 3.6 | 25.2 ± 3.7 | 0.61 | 25.6 ± 3.5 | 25.5 ± 3.5 | 0.63 |
| **Comorbidity** |  |  |  |  |  |  |
| Hypertension, n (%) | 9 (3.3) | 8 (2.9) | 0.92 | 13 (2.2) | 13 (2.2) | 0.30 |
| Heart disease, n (%) | 56 (20.5) | 57 (20.9) | 0.81 | 142 (24.3) | 127 (21.7) | 1 |
| Diabetes mellitus, n (%) | 1 (0.4) | 3 (1.1) | 0.06 | 5 (0.9) | 4 (0.7) | 0.37 |
| **Alcohol consumption** |  |  | 0.32 |  |  | 0.74 |
| Never, n (%) | 29 (10.6) | 34 (12.5) |  | 74 (12.7) | 69 (11.8) |  |
| Abstained, n (%) | 119 (43.6) | 116 (42.5) |  | 242 (41.4) | 220 (37.7) |  |
| 1–2 drinks/week, n (%) | 16 (5.9) | 14 (5.1) |  | 26 (4.5) | 19 (3.3) |  |
| 3–4 drinks/week, n (%) | 71 (26.0) | 67 (24.5) |  | 142 (24.3) | 183 (31.3) |  |
| Daily, n (%) | 25 (9.2) | 26 (9.5) |  | 73 (12.5) | 66 (11.3) |  |
| Missing data, n (%) | 13 (4.8) | 16 (5.9) |  | 27 (4.6) | 27 (4.6) |  |
| **Cigarette smoking** |  |  | 0.86 |  |  | 0.99 |
| Never, n (%) | 35 (12.8) | 35 (12.8) |  | 84 (14.4) | 83 (14.2) |  |
| Abstained, n (%) | 108 (39.6) | 110 (40.3) |  | 235 (40.2) | 227 (38.9) |  |
| Occasionally, n (%) | 41 (15.0) | 32 (11.7) |  | 71 (12.2) | 71 (12.2) |  |
| Often, n (%) | 10 (3.7) | 13 (4.8) |  | 23 (3.9) | 25 (4.3) |  |
| Daily, n (%) | 27 (9.9) | 25 (9.2) |  | 52 (8.9) | 52 (8.9) |  |
| Missing data, n (%) | 52 (19.0) | 58 (21.2) |  | 119 (20.4) | 126 (21.6) |  |
| **Smoking amount** |  |  | 0.96 |  |  | 0.17 |
| None or missing, n (%) | 159 (58.2) | 156 (57.1) |  | 335 (57.4) | 337 (57.7) |  |
| <5 cigarettes per day, n (%) | 14 (5.1) | 16 (5.9) |  | 32 (5.5) | 32 (5.5) |  |
| 5–10 cigarettes per day, n (%) | 22 (8.1) | 19 (7.0) |  | 35 (6.0) | 40 (6.8) |  |
| 11–19 cigarettes per day, n (%) | 52 (19.0) | 51 (18.7) |  | 123 (21.1) | 116 (19.9) |  |
| 1–2 packs per day, n (%) | 25 (9.2) | 31 (11.4) |  | 59 (10.1) | 58 (9.9) |  |
| >2 packs per day, n (%) | 1 (0.4) | 0 (0.0) |  | 0 (0.0) | 1 (0.2) |  |

Abbreviations:

Allopurinol (+): hyperuricemic patients treated with allopurinol only (n = 273)

Benzbromarone (+): hyperuricemic patients treated with benzbromarone only (n = 584)

ULT (–): cohorts of non-ULT users matched 1:1 by propensity score and the index date of ULT prescription to case cohorts of ULT users of allopurinol (+) (n = 273) and benzbromarone (+) (n = 584)
