# Supplementary material for: Effect of Urate-Lowering Therapy on All-Cause and Cardiovascular Mortality in Hyperuricemic Patients without Gout: A Case-Matched Cohort Study
Source: PLoS One. 2015 Dec 18;10(12):e0145193. doi: 10.1371/journal.pone.0145193 (PMC4684295; doi:10.1371/journal.pone.0145193)
Supplement: S3 Table — (DOCX) [file pone.0145193.s005.docx]

**S3 Table.** Mortality risk according to the presence of ULT use in subgroups stratified by concomitant medications and by duration of ULT use

| **Asymptomatic** | **ULT** |  |  | **All-cause mortality** | | | | |
| --- | --- | --- | --- | --- | --- | --- | --- | --- |
| **Hyperuricemic Patients** |  | **Number** | **PY** | **Event number** | **Mortality (10^−3^ PY)** | **MRR** | **HR (95% CI)** | *p* |
| ***Concomitant drugs*** |  |  |  |  |  |  |  |  |
| Anti-hypertensive (–) | – | 303 | 1955.64 | 7 | 3.58 |  | Reference |  |
|  | + | 303 | 1947.41 | 7 | 3.59 | 1 | 1.99 (0.41–1.59) | 0.40 |
| Anti-hypertensive (+) | – | 721 | 4509.63 | 64 | 14.19 |  | Reference |  |
|  | + | 721 | 4618.01 | 38 | 8.23 | 0.58* | 0.53 (0.35–0.81) | <0.01 |
| Anti-diabetic (–) | – | 872 | 5532.91 | 52 | 9.40 |  | Reference |  |
|  | + | 872 | 5593.23 | 36 | 6.44 | 0.69 | 0.75 (0.47–1.19) | 0.22 |
| Anti-diabetic (+) | – | 152 | 932.35 | 19 | 20.38 |  | Reference |  |
|  | + | 152 | 972.19 | 9 | 9.26 | 0.45 | 0.82 (0.17–3.88) | 0.80 |
| Lipid-lowering agent (–) | – | 860 | 5447.82 | 56 | 10.28 |  | Reference |  |
|  | + | 860 | 5498.63 | 40 | 7.27 | 0.71* | 0.59 (0.39–0.89) | 0.01 |
| Lipid-lowering agent (+) | – | 164 | 1017.44 | 15 | 14.74 |  | Reference |  |
|  | + | 164 | 1066.49 | 5 | 4.69 | 0.32 | 0.33 (0.04–2.86) | 0.32 |
| ***Duration of ULT use**** |  |  |  |  |  |  |  |  |
| <60 days | – | 497 | 3153.75 | 29 | 9.20 |  | Reference |  |
|  | + | 497 | 3166.00 | 26 | 8.21 | 0.89 | 0.83 (0.49–1.42) | 0.50 |
| 60–364 days | – | 218 | 1371.51 | 16 | 11.67 |  | Reference |  |
|  | + | 218 | 1397.14 | 10 | 7.16 | 0.61 | 0.60 (0.27–1.34) | 0.22 |
| 1–2 years | – | 118 | 743.73 | 8 | 10.76 |  | Reference |  |
|  | + | 118 | 750.01 | 6 | 8.00 | 0.75 | 0.73 (0.25–2.15) | 0.57 |
| >2 years | – | 191 | 1196.27 | 18 | 15.05 |  | Reference |  |
|  | + | 191 | 1252.26 | 3 | 2.40 | 0.16* | 0.15 (0.05–0.53) | <0.01 |

^a^HRs were adjusted for the propensity score. **p* < 0.05

Abbreviations: ULT: urate lowering therapy; PY: person-years; MRR: mortality rate ratio; HR (95% CI): hazard ratio (95% confidence interval)
