# Supplementary material for: Effect of Urate-Lowering Therapy on All-Cause and Cardiovascular Mortality in Hyperuricemic Patients without Gout: A Case-Matched Cohort Study
Source: PLoS One. 2015 Dec 18;10(12):e0145193. doi: 10.1371/journal.pone.0145193 (PMC4684295; doi:10.1371/journal.pone.0145193)
Supplement: S1 Text — (DOCX) [file pone.0145193.s006.docx]

**S1 Text. Data Availability Statement**

All data and related metadata were deposited in an appropriate public repository. The data on the study population that were linked to the NHIRD (<http://w3.nhri.org.tw/nhird//date_01.html>) are maintained in the NHIRD (<http://nhird.nhri.org.tw/>). The NHRI is a nonprofit foundation established by the government. These data were released by the NHIRD for research uses. Every interested researcher is able to obtain the data in the same way that we did.
